# Still Living Better through Chemistry: An Update on Caloric Restriction and Caloric Restriction Mimetics as Tools to Promote Health and Lifespan

**DOI:** 10.3390/ijms21239220

**Published:** 2020-12-03

**Authors:** Carla Almendáriz-Palacios, Darrell D. Mousseau, Christopher H. Eskiw, Zoe E. Gillespie

**Affiliations:** 1 Department of Food and Bioproduct Sciences, University of Saskatchewan, Saskatoon, SK S7N 4A8, Canada; c.almendariz@usask.ca (C.A.-P.); c.eskiw@usask.ca (C.H.E.); 2 Cell Signalling Laboratory, Department of Psychiatry, University of Saskatchewan, Saskatoon, SK S7N 5E5, Canada; darrell.mousseau@usask.ca; 3 Department of Biochemistry, Microbiology and Immunology, University of Saskatchewan, Saskatoon, SK S7N 5E5, Canada

**Keywords:** caloric restriction, caloric restriction mimetics, amino acid restriction, lifespan, healthspan, mammalian target of rapamycin (mTOR), general control nonderepressible 2 (GCN2)

## Abstract

Caloric restriction (CR), the reduction of caloric intake without inducing malnutrition, is the most reproducible method of extending health and lifespan across numerous organisms, including humans. However, with nearly one-third of the world’s population overweight, it is obvious that caloric restriction approaches are difficult for individuals to achieve. Therefore, identifying compounds that mimic CR is desirable to promote longer, healthier lifespans without the rigors of restricting diet. Many compounds, such as rapamycin (and its derivatives), metformin, or other naturally occurring products in our diets (nutraceuticals), induce CR-like states in laboratory models. An alternative to CR is the removal of specific elements (such as individual amino acids) from the diet. Despite our increasing knowledge of the multitude of CR approaches and CR mimetics, the extent to which these strategies overlap mechanistically remains unclear. Here we provide an update of CR and CR mimetic research, summarizing mechanisms by which these strategies influence genome function required to treat age-related pathologies and identify the molecular fountain of youth.

## 1. Introduction

Aging is an inevitable process experienced by all life on this planet. In humans (and numerous other species), aging comes with a battery of age-linked pathologies, including type 2 diabetes (T2D), cardiovascular disease (CVD), atherosclerosis, osteoarthritis, neurodegenerative diseases (ND), and cancer [1,2,3,4]. These pathologies are often the combination of nature and nurture; genetics play an important role, but environmental conditions (including exercise and diet) also have a significant impact on the rate at which individuals acquire disease and age. Excessive calorie intake increases the risk of many of the aforementioned age-linked pathologies, deleteriously promoting chronic inflammation and signaling insensitivities. Does aging have to be fraught with deteriorating health and increasingly poor quality of life until death? Or could healthy aging be feasible? Those that exercise and have a moderate diet are already shown to be at lower risk of numerous diseases, but could this effect be enhanced to promote longer, healthier lives? Addressing this question could have a tremendous societal impact, decreasing the socioeconomic burden of an aging population and, as a result, is an intensively researched area of biology.

A key area of research for maintaining longer, healthier lives is caloric restriction (CR). CR is the reduction of nutritional intake to 60–80% ad libitum without inducing malnutrition or starvation. This approach has been successful and reproducible across laboratory-model organisms in extending lifespan (from yeast to nonhuman primates) [5,6,7,8,9]. Short-term trials of CR in humans have also proven effective in preventing and improving the treatment outcomes of age-linked pathologies such as cancer [10,11]. Despite the promise of CR, this regime is unlikely to be achieved, particularly as large percentages of individuals already struggle with obesity and thus moderating their diets (World Health Organization, 2020). Therefore, the question is: could alternative dietary regimes that may not require decreased food consumption achieve the same beneficial effects as CR? Or is there a pharmacological mimetic of CR, a “magic pill,” that will alter how the body senses energy inputs and reproduces a CR-like state? 

Amino acid restriction (AAR) is a potential CR alternative, with current work focusing on the efficacy of total AAR or the restriction of specific amino acids in promoting health and lifespan [12,13]. Similarly, the immunosuppressant rapamycin [14] (and the structurally related family of compounds called rapalogs [15]), the polyphenol resveratrol (found in the skin of red fruits, such as grapes) [16,17], and the T2D treatment metformin [18,19] have been examined for their ability to reproduce CR-like effects without dietary alterations. Although it is generally accepted that these compounds can promote health and lifespan, exactly how this is achieved is still being investigated. Therefore, it is necessary to understand how these compounds modulate cell function to better comprehend the aging process but also to identify other potential pro-longevity strategies. Here, we will discuss the theories of aging in relation to CR and how CR is sensed at the cellular level before discussing AAR and potential CR mimetics (CRMs) in the same contexts. 

## 2. Caloric Restriction Attenuates Features and Processes of Cellular Aging

Aging is caused by the accumulation of numerous cellular alterations that induce cells to senescence, which consequently secrete inflammatory proteins that spread this state in neighboring cells. The accumulation of senescent cells contributes to age-associated diseases, and the clearance of these cells has been shown to delay or improve age-related phenotypes [20,21,22]. Key alterations associated with promoting aging (including the accumulation of senescent cells) include increased genomic instability, telomere shortening, changes in the epigenome, loss of protein homeostasis (proteostasis), deregulated nutrient sensing, mitochondrial dysfunction, stem cell exhaustion, and altered intracellular signaling [23]. CR has shown promise in attenuating age-promoting factors, reducing the number of senescent cells in a culture and model organisms [24]. CR was first reported in 1935 [5] and since then has become the most reproducible and reliable method of extending health and lifespan across model organisms, ranging from unicellular to nonhuman primates (yeast, [9], fruit flies [25], nematodes [26], crustaceans [27,28], spiders [29], lab mouse/rats [30], and monkeys [6,8,31]. At the molecular level, CR mitigates and reverses features of cellular aging. The free-radical theory of aging proposes that increased energy intake can result in more ROS as a result of oxidative phosphorylation in the mitochondria, contributing to lipid, protein, and DNA damage and advancing age (reviewed in [32,33]). CR counteracts this by lowering metabolic rates [34,35], improving DNA damage repair [36,37,38,39,40], and decreasing oxidative stress [41,42,43]. Although this theory is generally accepted, free-radical production and oxidative stress are not always decreased in response to CR [44,45]; however, increased expression of antioxidant enzymes may act as a compensatory mechanism for this.

In connection with ROS and mitochondrial dysfunction, the insulin sensitivity of cells decreases with age, increasing levels of serum glucose and insulin required for cellular responses. As a result, glucose metabolism is upregulated, increasing cell replication, decreasing the time between cell divisions, and permitting less time for the maintenance and repair of DNA [46,47]. Together, these processes push cells toward senescence. CR decreases levels of circulating glucose and insulin, increases insulin sensitivity, and extends health and lifespan [48,49,50,51]. The upregulation of insulin-like growth factor-1 (IGF-1) also occurs with age, again promoting cell growth, division, and metabolism. CR decreases the activity of the IGF-1 signaling pathway, promoting cellular maintenance, and repair in mice [52]. However, human clinical trial data have been less consistent, with two-year CR having no impact on circulating IGF-1 levels [53,54]. Despite these findings, alternative analyses of the same trial concluded that CR decreased the rate of biological aging [55].

The hormesis model of aging proposes that low levels of stress primes cells, enabling them to prepare cellular processes required to appropriately counteract larger stress [56]. CR increases the expression of proteins involved with DNA repair, free-radical scavenging, and stress, as well as mediating ROS-producing pathways [24,40,42,43]. This model is often favored for explaining the health and lifespan benefits of CR, whereby CR activates transcription factors and mechanisms involved in regulating gene expression and protein translation to better mediate cellular stress responses [57,58] (reviewed in [59]). 

### 2.1. How are Nutrients Sensed at the Cellular Level? 

Key in promoting health and lifespan at the cellular and organismal level is the manipulation of how nutrients are detected. The target of the rapamycin (TOR) pathway is a highly conserved nutrient-sensing pathway comprising multiple proteins [60]. In mammals, the mammalian (m)TOR signaling cascade indirectly detects inputs from multiple sources, including growth signals, amino acids, and energy levels. There are two mTOR complexes: mTOR complex 1 (mTORC1) and mTOR complex 2 (mTORC2). mTORC1 is directly involved in mediating cellular responses to nutrients, although recently, mTORC2 has also been shown as nutrient-sensitive [61]. Multiple growth factor pathways (such as the insulin/IGF-1 pathway) converge upstream of mTORC1 at the tuberous sclerosis complex (TSC; a heterotrimeric complex of TSC1, TSC2, and TBC1D7 [62]). In the presence of growth factors, the phosphoinositide 3-kinase/3-phosphoinositide-dependent protein kinase 1/protein kinase B (PI3K/PDK1/AKT) pathway inhibits TSC by the AKT-dependent multisite phosphorylation of TSC2. This induces the dissociation of TSC from the lysosomal membrane [63], inactivating the RAS homolog enriched in brain (Rheb), which is also partially localized to the lysosome. Subsequently, mTORC1 is phosphorylated and activated by Rheb, promoting downstream cellular growth and proliferation (reviewed by [64]). Other growth factors functioning via TSC inhibition to activate mTORC1 include receptor tyrosine kinase-dependent Ras signaling, wingless-related integration site (WNT), and tumor necrosis factor α (TNFα) pathways (reviewed in [64,65]). Exactly how the TSC integrates the input of these various signals is unknown. When active, mTORC1 promotes various processes, including nucleotide, lipid, and protein synthesis, which stimulate cell growth and proliferation. In the absence of growth factors, TSC sequesters Rheb at the lysosomal membrane [66,67], inhibiting mTORC1, and shifting cellular processes toward maintenance and repair, upregulating autophagy (recycling of cellular components) and inhibiting the synthesis of new molecules.

### 2.2. What Downstream Effects Does Caloric Restriction Have on Genome Function?

CR does not directly inhibit mTORC1 but modulates upstream energy-sensing complexes. Adenosine monophosphate kinase (AMPK) is activated (phosphorylated by liver kinase B1; LKB1) in response to increased AMP/ATP ratios, whereas Sirtuin 1 (SIRT1) is activated by increased NAD^+^ levels, indicating low cellular energy availability [68,69,70]. AMPK and SIRT1 also activate one another, although AMPK is active much sooner (seconds/minutes) following CR when compared to SIRT1 activation (4–12 h) [71]. Perhaps SIRT1 functions later in response to CR in order to maintain AMPK activity. AMPK is also regulated by increases in intracellular Ca^2+^ (likely the result of hormonal changes), specifically via phosphorylation by calcium/calmodulin-dependent protein kinase kinase B (CamKKB) [72,73]. Once activated, SIRT1 deacetylates downstream targets such as p53, inhibiting p53-mediated gene expression [74,75]. However, there is potential for additional feedback as SIRT1 expression depends on interaction with Forkhead Box O3 (FOXO3a) and p53 under CR, whereas FOXO family members are also involved in regulating IGF-1 signaling upstream of mTOR [70,76]. Moreover, SIRT1 deacetylates components of nuclear factor (NF)-κB to decrease the transcription of proinflammatory genes [77,78,79,80]. SIRT1 also deacetylates other mTOR-linked proteins, such as hypoxia-inducible factor 1α (HIF1α) [81,82], which is usually increased with age [83,84,85]. The deacetylation of HIF1α decreases the expression of genes involved in cell cycle progression, angiogenesis, and glucose metabolism [86]. 

The impact of complex signaling in response to CR and how it could potentially reverse age-linked transcriptomic changes have been examined through numerous transcriptome-wide studies across model organisms, including humans [87,88,89,90,91,92,93,94,95]. In general, whole-genome studies (either by microarray or RNA sequencing) have concluded that CR was able to upregulate “antiaging” genes, including genes that inhibit oxidative stress, decrease age-linked inflammation, decrease metabolism, and decrease genes involved in pathways such as IGF-1 signaling and cell cycle progression (Table 1). These findings are striking given the consensus of several publications linking pathways associated with aging to modulation by CR. Furthermore, these data highlight the importance of age at CR initiation and CR duration. For example, short-term CR induces different transcriptome profiles to long-term CR [96,97], whereas the age of CR onset also impacts its efficacy [98,99]. Analyses have been conducted over a period spanning two decades; therefore, in addition to the promising results and concordant conclusions, additional meta-analyses of datasets may provide new insights into processes governing the progression of aging as knowledge of genes and pathways evolves. 

Aging is the result of numerous genetic and environmental factors that may not all be controllable in laboratory experiments. Furthermore, tissues and organs exhibit unique aging processes [100]. For example, some tissues age faster and secrete substantially more inflammatory factors. Mitochondria-rich tissues may exhibit greater susceptibility to DNA-damage-linked aging, whereas other tissues respond more to the loss of insulin and IGF-1 sensitivity. Recently, Ma and colleagues examined seven different tissues from rats for age-linked changes and their potential for reversal by CR from midlife [101]. Ma et al. also examined the composition of cell types, determining that, in accordance with changes in gene expression linked to inflammation/the immune system, cell-type composition within the rat tissues changed. Although CR rescued many age-associated changes at the genomic level, there were also CR-induced gene expression changes unique to CR in each tissue [101], corroborating other studies in mouse liver and flies [97,102]. In addition to tissue-specific changes, treatment length and dosage effects need to be considered, given that chronic or more stringent CR potentially has a different impact than shorter-term or less stringent regimes (Table 1). 

Although understanding the aging transcriptome has progressed to the development of a transcriptomic clock (software that can predict tissue age based on transcript profiles), mechanisms behind these changes are still lacking, and pathway analyses are still vague [103]. It is important to go beyond the aging transcriptome and the impact of CR to understand how other regulatory elements of the genome are influenced. This information will further aid in the understanding of potential CR mimetics, their similarity to CR, and their ability to increase health and lifespan. 

## 3. Amino Acid Restriction: An Alternative to Caloric Restriction?

Although CR has been studied for over a century, removing specific elements of diets is a more recently evolved area of longevity research. Amino acid restriction (AAR) centers around the theory that it is not CR per se that is responsible for increasing health and lifespan but the reduction of amino acids specifically from the diet. This consideration, combined with biochemical evidence that the presence/absence of amino acids alone is sufficient to modulate mTORC1 function [104,105,106,107], supports the proposal that AAR could be an alternative to CR.

### 3.1. Does Amino Acid Restriction Extend Health and Lifespan? 

The restriction of all amino acids has been shown to extend lifespan in yeast [9,108], ants [109], and flies [108,110]. Additionally, the restriction of specific amino acids, such as branched-chain amino acids (BCAAs) in mice, can be as beneficial as total amino acid restriction [111], whereas in rats, the restriction of individual essential amino acids (leucine, lysine, methionine, and threonine) and total AAR reduced IGF-1 levels [112]. Tryptophan restriction increased the lifespan of rodents [113]; however, not all AAR is successful, with glutamic acid deprivation decreasing yeast lifespan [114]. Leucine restriction improves metabolic health, glucose tolerance, and insulin sensing in mice [115,116]. Protein restriction prior to surgery exhibited physiological stress protection in mice [113], whereas low-protein diets in humans under 65 years of age resulted in an overall reduction in IGF-1 levels, cancer, and mortality [117]. Furthermore, high-protein diets are linked with higher mortality rates when compared to diets based on low-carbohydrate and high vegetable content [118]. There is also an increased association with red meat and diabetes, cancer, and ischemic heart disease [119,120], whereas reduction in protein intake reduces circulating IGF-1 levels [54]. AAR-linked reductions in IGF-1 levels and improved glucose sensitivity are parallel to those observed in CR and important in extending health and lifespan. The AAR of specific amino acids further influences the post-translational modification of proteins. For example, BCAAs are important in the formation of acetic acid, a molecule used in the acetylation of histones, as well as in the regulation of autophagy, supporting findings of BCAAs in promoting lifespan-extending biological states [111,112,121]. This effect on epigenetic modification is not unique to BCAAs, with a decrease in methionine leading to decreased *S*-adenosyl-methionine (SAM) and reduced histone methylation [122]. Therefore, the combination of amino acids in AAR is likely important in directing which pathways are influenced within an organism and subsequent genomic and epigenomic responses. It is likely many of the beneficial impacts of total AAR, or the restriction of specific amino acids, are the result of mTORC1 inhibition and upregulated autophagy. AAR has been shown to upregulate autophagosomal–lysosomal pathways, promoting the degradation of cytoplasmic proteins and organelles, thereby providing free amino acids for protein synthesis [123,124]. 

Unlike CR, AAR is more complex and can be the result of restricting single or multiple amino acids. AAR strategies focusing on methionine restriction are the best defined to-date; however, this is only effective if cystine is also restricted and is therefore referred to as sulfur amino acid restriction (SAAR). In addition to increasing lifespan [114,125,126,127], SAAR lowers levels of serum IGF-1, insulin, glucose, and thyroid hormone, increasing insulin sensitivity, decreasing body weight, improving resistance to oxidative stress in various mice and rat strains [128,129,130,131,132,133], and decreasing mitochondrial ROS production and protein oxidation [134,135]. Additionally, in mice, SAAR prevents the onset of T2D [136] and extends the lifespan of progeroid-models [137,138]. Therefore, as with CR, SAAR counteracts many of the molecular processes associated with aging.

Although AAR is capable of extending health and lifespan, the deprivation of different amino acids differentially impacts the transcriptome. When compared to 15 other amino acids in breast and prostate cancer cells, the restriction of methionine was able to elicit the largest transcriptional changes, likely due in part to additional impacts on the epigenome [122]. As cancer cells are often hyperproliferative and have dysregulated cell cycles, to better understand the impact of AAR in aging, the replication of these studies in noncancerous human cell lines would be beneficial. Evidence for amino-acid-specific effects is provided by methionine restriction, which extends the lifespan of *Drosophila melanogaster*, albeit not to the same degree as total AAR [108]. Similarly, leucine restriction exerts similar benefits as methionine restriction (although not as consistently) [116]. In flies, the restriction of tryptophan or arginine required the general control nonderepressible 2 (GCN2) protein (a protein kinase that specifically responds to amino acid levels) to achieve health-promoting effects, whereas others (histidine and arginine) did not [139]. Finally, protein-restricted mice supplemented with different BCAAs exhibited varied metabolic profiles. [111,140]. This may be due to the different sensitivity of mTORC1 to each amino acid, in particular leucine and arginine [104]. Although there is much evidence regarding the benefits of either total AAR or SAAR restriction, it will be important to determine how healthy tissues and organisms respond to the removal of individual amino acids. Furthermore, understanding genomic changes and mechanisms mediating these processes will aid in the development of diets in which amino acid levels can be manipulated to treat many age-linked diseases without reducing overall caloric intake. 

### 3.2. Is Amino Acid Sensing also Regulated through mTOR?

Although some organisms can synthesize all amino acids, mammals must rely on extracellular sources for a subset of amino acids (termed essential amino acids). Amino acid levels can also be used to modulate mTOR activity to direct subsequent cellular processes [104,105,106,107]. Amino acid sensing by mTOR is complex, with potentially distinct upstream receptors for each amino acid. Few of these have been identified; however, in general, amino acid depletion downregulates mTORC1, resulting in decreased protein synthesis and cell proliferation. This inactivation also promotes autophagy, catabolizing pre-existing molecules for the amino acids required to prevent starvation [141]. If enough autophagy occurs, mTORC1 is upregulated. 

mTORC1 is localized to the lysosomal surface in the presence of amino acids. In general, amino acid sensors indirectly promote stable dimerization between RagA/B and RagC/D, which are targeted to the lysosome via Ragulator (as the RagA/B/C/D GTPases do not have a lipid-targeting signal, this acts as an intermediate). To interact with mTORC1 (via the raptor subunit), RagA/B must be GTP loaded and RagC/D must be GDP loaded. Rheb then activates mTOR (mTORC1) in a Rag-dependent manner [142,143,144]. If artificially anchored to the lysosome, mTORC1 becomes insensitive to amino acids while still responding to insulin levels [142]. Therefore, it is likely that Rheb and Rag form part of a regulatory network that enables appropriate mTORC1 activation in response to a balance of amino acids and growth factors.

**Figure 1:**
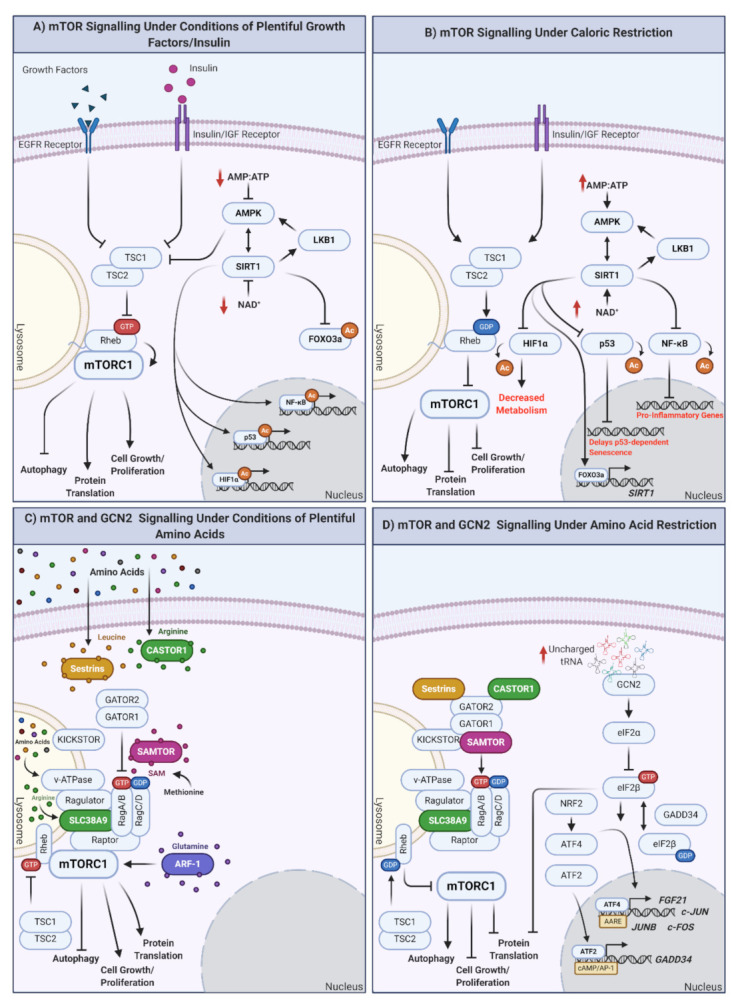
How are nutrients sensed at the cellular level? The mTOR and GCN2 pathways. (**A**) mTOR signaling in response to plentiful nutrient (growth hormone, insulin) supply. Under conditions of plentiful nutrients, growth factors and insulin inhibit TSC1/2, leading to the inhibition of Rheb and the induction of mTORC1 at the lysosomal surface. mTORC1 upregulation results in autophagy inhibition and the promotion of protein translation, cell growth, and proliferation. Under these conditions, cellular ATP levels increase, reflective of plentiful nutrient status. ATP increase inhibits AMPK, modulating the AMPK-SIRT1-LKB1 feedback loop and inducing mTORC1. Independently of mTORC1, SIRT1 also downregulates the expression of FOXO3a and promotes the synthesis of genes/proteins involved in the cell cycle, as well as response inflammation and cellular stress (when required). (**B**) mTOR signaling under conditions of caloric restriction. In the absence of nutrients (or under conditions of caloric restriction; CR) TSC1/2 are activated, Rheb is GDP loaded, and mTORC1 is inhibited by undocking from the lysosomal surface. This promotes autophagy and inhibits protein translation, cell growth, and proliferation. Simultaneously, ATP levels decrease, increasing the AMP/ATP ratio indicative of low cellular energy status, activating AMPK, and modulating the AMPK-SIRT1-LKB1 feedback loop. In addition, proinflammatory transcription/translation is inhibited, metabolism is decreased, and longevity promoting FOXO3a is translocated to the nucleus to regulate its target genes. (**C**) mTOR signaling under plentiful amino acid availability. In the presence of amino acids, mTORC1 integrates signals from multiple amino acid receptors: (inhibited sestrin (leucine), castor (arginine), or samtor (methionine) complex formation), glutamine sensor ARF-1, lysosomal arginine sensor SLC38A9, and lysosomal amino acid sensor v-ATPase. In the presence of all amino acids, mTORC1 is active and promotes cellular growth and proliferation. If individual amino acids are absent, this could result in mTORC1 inhibition and shift cells to a status of amino acid restriction (AAR). (**D**) mTOR and GCN2 under conditions of amino acid restriction (AAR). In the absence of amino acids, mTORC1 is inhibited, promoting the previously mentioned prolongevity factors, including autophagy and preventing cell proliferation. GCN2 is activated under conditions of AAR by the binding of uncharged tRNAs. eIF2α is then converted to eIF2β, which cycles between GTP- and GDP-loaded states, regulated by GADD34. eIF2B promotes translocation of ATF4 to the nucleus, where it regulates subsequent expression of genes with amino acid response elements (AARE). NRF2 is also upregulated in response to AAR and can promote upregulation of ATF4. ATF2 similarly binds cAMP/AP-1 motifs to promote the expression of GADD34. Created with BioRender.com.

Upstream of the Rag GTPases are cellular amino acid sensors. Of these, the sensors for arginine and leucine (sestrin2 and cytosolic arginine sensor for mTORC1 (CASTOR)) [145,146,147,148] bind their respective amino acids, disrupting interactions with and activating GTPase-activating protein toward Rags (GATOR)2, inhibiting GATOR1, and activating RagA/B and, ultimately, mTORC1 [145,149,150] (Figure 1C). In the absence of arginine/leucine, their respective sensors are bound to GATOR2, inhibiting it as well as mTORC1 (Figure 1D). Unlike arginine/leucine, methionine is sensed by the S-adenysyl-methionine (SAM) sensor (SAMTOR) via the presence of its metabolite SAM. SAM binds SAMTOR, dissociating it from GATOR1. Amino acids are also sensed from the lysosome; for example, SLC39A9 is a lysosomal arginine sensor that interacts with the Rag-GTPase-Ragulator-v-ATPase complex to activate mTORC1 (reviewed by [60]). This process may couple the release of essential amino acids from the lysosome to activation of mTORC1 and cell growth. 

Beyond utilizing specific amino acid receptors, mTORC1 also responds to amino acid signaling via Rag-GTPase-independent methods. Glutamine requires lysosomal H^+^ATPase (v-ATPase) and vesicle trafficking ADP ribosylation factor (Arf-1 GTPase) [151,152]. Glutamine has also been reported to activate mTORC1 through a Rag-dependent mechanism [153]. The ability of AAR to modulate mTOR signaling to inhibit mTORC1 and, subsequently, upregulate similar downstream processes as seen with CR, makes it a strong candidate as an alternative to CR. 

## 4. Are Amino Acid Restriction and Caloric Restriction the Same? 

In addition to modulating mTORC1 function, AAR also mediates mTORC1-independent functions via the integrated stress response (ISR). The ISR rapidly assimilates extracellular stress signals, such as AAR, through protein kinases that function to prevent the exhaustion of cellular resources. This process also likely occurs in response to CR, thereby reducing numerous energy status indicators, including levels of amino acids and growth factors. As intracellular amino acid levels decrease, so do levels of charged tRNA. Uncharged tRNA then bind and activate GCN2, which then phosphorylates eukaryotic initiation factor 2 (eIF2)α, which is converted into an inhibitor of eIF2β. The inhibition of eIF2β will slow the rate at which eIF2α is reloaded with GTP, ultimately reducing GTP-GDP exchange rates on eIF2α, necessary for mRNA translation re-initiation and decreasing global translation. Although global translation is decreased, the translation of specific transcription factors such as activating transcription factor (ATF) 2, ATF4, ATF5, and growth arrest and DNA -damage-inducible protein (GADD34) are increased [154] (reviewed by [155]). ATF4 translocates to the nucleus where it binds amino acid response elements (AAREs), upregulating the expression of amino acid transporters, metabolic regulators, antioxidant defenses, and other homeostatic processes to promote the re-establishment of homeostatic cellular conditions. GADD34 enables feedback signaling to permit the translation of upregulated stress-response genes [156]. Some ATF4-regulated genes require the phosphorylation of ATF2 to upregulate transcription. ATF2 specifically targets genes that do not respond to other amino acid response linked transcription factors [157]. Therefore, AAR can be regulated independently of the mTOR pathway. In addition, GCN2 is required to extend lifespan in yeast and flies in response to AAR [114,158], with GCN2-deficient organisms displaying aging phenotypes. As downstream processes regulated by GCN2 and mTORC1 significantly reduce protein translation, this is one potential mechanism by which longevity is promoted. GCN2 signaling is responsive to CR likely through a decrease in amino acids and therefore plays an important role in mediating the response to CR, albeit more specific to amino acid sensing. The role of mTORC1 in response to AAR and CR has been extensively examined; however, the role of GCN2 in promoting health and lifespan remains relatively unexplored.

The IRS and mTOR pathways feedback on one another, linking cellular responses to CR and AAR in promoting health and lifespan. One important molecule involved in this feedback is fibroblast growth factor 21 (FGF21), which is upregulated by ATF2/4. The FGF21 promoter contains an AARE [159,160], and FGF21 is implicated in promoting the beneficial effects of CR and AAR/SAAR [161,162]. Additionally, the overexpression of FGF21 increases lifespan [163] and prevents cellular senescence via SIRT1 [164], demonstrating its significant role in longevity and well-established longevity-promoting pathways. Counterintuitively to its upregulation in response to CR and AAR, FGF21 levels are also increased with age [165,166,167]. It has been proposed that this could be due to insensitivity to FGF21 levels/loss of FGF21 receptors [166,168,169]; however, findings are mixed [167,170]. Although the reason for an increase in FGF21 levels in aging, as well as in response to CR, is unclear, there is a consensus that this molecule upregulates metabolism and reduces the impacts of chronic disease (reviewed by [171]) by preventing insulin resistance ([172,173]). FGF21 also activates the mTORC1-S6K axis via mitogen activator protein kinase (MAPK), perhaps as the result of feedback signaling from increased amino acid availability due to autophagy, with increased mTORC1 levels demonstrated to mediate the beneficial impacts of FGF21 in vitro [174]. As a molecule primarily secreted from the liver, FGF21 could act as a signal of global energy status across many tissues, promoting energy-saving states in other cells. 

## 5. Caloric Restriction Mimetics

CR clearly has positive impacts on health and lifespan. However, with 39% of the world’s population already overweight (World Health Organization, 2020), this is a difficult strategy to implement. Alternatively, a variety of compounds exist that mimic CR without having to restrict food intake. Caloric restriction mimetics (CRMs) often function via similar pathways to CR, such as the mTOR signaling cascade, and have the potential to be used as treatments to prevent the development of age-related pathologies and promote healthy aging. 

### 5.1. Rapamycin

**Figure 2:**
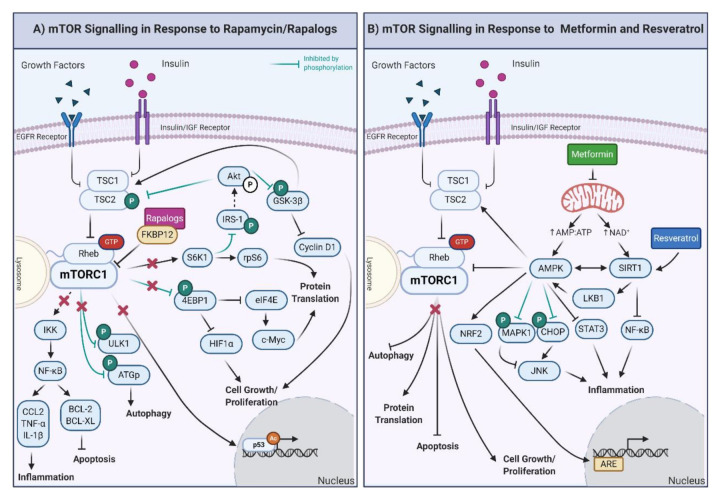
(**A**) Downstream effects of mTOR signaling in response to rapamycin and rapalogs. The inhibition of mTORC1 results in the decrease of the inflammatory response and promotion of apoptosis through regulation of IκB kinase. ULK1 is also inhibited, promoting autophagy. Furthermore, the activity of the eukaryotic translation initiation factor 4E-binding protein 1 (4EBP1) and S6K1 proteins is decreased, which results in diminished cell growth, proliferation, and protein translation. The inhibition of mTORC1 also decreases p53-mediated gene expression. Moreover, through the inhibition of S6K1, Akt activity is upregulated, generating a feedback loop to the products of decreased mTORC1 activity, specifically via the inhibition of TSC1/2 and GSK-3β. IRS-1: insulin receptor substrate 1; CCL-2: C-C motif chemokine ligand 2, BCL-2: B-cell lymphoma 2; BCL-XL: B-cell lymphoma-extra-large. (**B**) Downstream effects of mTOR, AMPK, and SIRT1 signaling in response to metformin and resveratrol. Metformin exposure decreases mitochondrial function, increasing the AMP/ATP ratio and NAD^+^ levels. The increased AMP/ATP ratio activates AMPK, which, in turn, decreases protein translation, cell growth/proliferation, and stimulates autophagy and apoptosis. AMP also activates the nuclear factor erythroid 2-related factor 2 (NRF2), which triggers the synthesis of genes involved in the antioxidant response (ARE). Through inhibition by phosphorylation of the mitogen-activated protein kinase (MAPK1) and CCAAT-enhancer-binding homologous protein, AMPK inhibits and activates the c-Jun N-terminal kinase (which is involved in inflammation, JNK). Furthermore, AMPK also reduces the inflammatory response through the inhibition of the signal transducer and activator of transcription (STAT3). In the presence of resveratrol, the most potent inducer of SIRT1, inflammatory proteins are decreased by the inhibition of NF-κB. Finally, AMPK is also activated by phosphorylation of the liver kinase B1 (LKB1) and deacetylation of SIRT1, creating a feedback loop. Created with BioRender.com.

Rapamycin, also known as sirolimus, was initially isolated in 1964 from soil containing *Streptomyces hygroscopicus* on Rapa Nui (Easter Island) during a Canadian expedition to the South Pacific. First described as an antifungal, the immunosuppressive and antitumor properties of rapamycin led to its frequent use in clinical settings [175]. In 2006, it was proposed that rapamycin could be used to slow aging and improve many physiological functions that deteriorate with age in humans, making this compound one of the first CRMs [176]. Rapamycin forms a complex with the FK506-binding protein 12 (FKBP12), which induces inhibition of mTORC1 and subsequently the mTOR pathway (Figure 2A) [177,178]. Similar to CR, the rapamycin-mediated inhibition of mTORC1 decreases cellular growth, proliferation, and protein translation [179,180,181] and upregulates autophagy [182,183], thus promoting health and lifespan. 

In addition to its lifespan-extending properties, additional studies have shown that rapamycin can ameliorate age-related disease phenotypes in cell-based models of cancer [184,185,186], CVD [187,188], premature aging diseases (e.g., HGPS [189,190,191]), and ND [192]. These findings can be recapitulated at the organismal level, including in ND (e.g., amyotrophic lateral sclerosis [193], tauopathies [194], Alzheimer’s disease [195,196,197], Parkinson’s disease [198]), cancer [199,200], obesity [201], disrupted circadian clock [202,203], and laminopathies [204,205]. Furthermore, rapamycin pretreatment improves the success of pancreatic islet cell engraftment through the inhibition of inflammatory chemokines in mice models and human patients with type I diabetes [206]. The results of these studies have encouraged the exploration of rapamycin in humans beyond its use as an immunosuppressant following organ transplantation [207,208,209]. For example, when applied topically, rapamycin can improve the clinical appearance of the skin [210], but, more importantly, it can prevent insulin resistance [211]. Therefore, like CR, rapamycin has numerous benefits in various disease states to promote healthier aging across species. 

There is evidence indicating that rapamycin in a bona fide CRM due to its induction of autophagy. This is supported by combinatorial studies of CR and rapamycin, in which the same pathway is targeted [212]. However, there is also evidence that, unlike CR, which improves insulin sensitivity and glucose tolerance, rapamycin promotes insulin resistance [213]. This could be the result of chronic rapamycin treatment, which induces starvation pseudodiabetes (SPD), also triggered in response to prolonged fasting [214,215]. It is possible that in models where rapamycin induces SPD, the dose is too high and mimics starvation rather than CR. To further support that rapamycin functions alternatively to CR, Unnikrishnan et al. [14] demonstrated that CR, in combination with rapamycin, improved glucose and insulin intolerance when compared to control or rapamycin alone, indicating that CR could alleviate the secondary effect of rapamycin on glucose metabolism Although this may be a cause for concern in considering rapamycin as a lifespan-promoting compound, or even a CRM, there are numerous experimental considerations. For example, the increase in insulin insensitivity is not observed in all mouse strains and treatment conditions are often not consistent across studies (e.g., dose and length). Nevertheless, rapamycin-fed model organisms frequently live longer, healthier lives. 

To establish whether rapamycin is a CRM, it is important to consider changes in specific genes that are markers of CRM function. *Cyclin D1*, a cell cycle marker, increased in response to both CR and rapamycin in mouse liver tissue. However, transcript levels of other proteins involved in the same cellular process, such as *p16*, *p21*, and *p53*, were decreased [212,216] (Table 2). Unnikrishnan and colleagues also identified transcriptomic changes in the genome of mouse livers under regimes of ad libitum, CR, and rapamycin treatment [14]. Rapamycin was found to have overlapping, but distinct, gene expression profiles to those of ad libitum or CR [217], supporting that rapamycin is not a CRM. This could also provide evidence that to achieve health and lifespan extension, a subset of transcriptomic changes in response to CR/AAR/CRM is required, and other changes may be condition- or compound-specific and not essential for the desired effects. Furthermore, when combined, rapamycin and CR have greater impacts on both the transcriptome and metabolome of mice than when applied separately [217], with <20% of mRNA transcripts shared by CR and rapamycin. Moreover, of genes changing by CR, 74% matched genes previously identified in response to CR (using the GenDR database), but only 7.5% of rapamycin-induced gene expression changes were found in the same database. In comparing rapamycin and CR in white adipose tissue (WAT) of mice, fewer transcripts changed in response to rapamycin [216]. Similarly, CR induced greater changes in the yeast transcriptome than rapamycin, with CR upregulating genes for autophagy and β-oxidation, whereas rapamycin increased the expression of enzymes involved in the degradation of energy-storage molecules [218]. Both rapamycin and CR had beneficial impacts on reproduction in mice (increased preservation of ovarian primordial follicular reserves); however, the two treatments again exhibited divergent metabolic effects. Regardless, both were associated with higher transcript levels encoding the pro-longevity factor FOXO3a [219]. Therefore, although CR and rapamycin both function to extend health and lifespan consistently across organisms, the impacts of these compounds are transcriptomically varied.

### 5.2. Rapalogs: Rapamycin Analogs 

Rapamycin has many benefits; however, its poor solubility, bioavailability, and pharmacokinetics, as well as side- and off-target effects [228], have led to the development of rapalogs. Rapalogs are synthetic analogs of rapamycin that aim to improve clinical outcomes of rapamycin, with fewer off-target effects. These rapalogs, including everolimus, temsirolimus, and ridaforolimus, have been examined to determine if they can also extend health and lifespan. 

Everolimus (RAD001) is an orally administered rapalog that has been reported to have the same therapeutic properties as rapamycin with higher thermal stability, greater solubility, and improved pharmacokinetics [229]. Everolimus binds to FKBP12, inhibiting mTORC1, and results in the downregulation of the PI3K/AKT/mTOR pathway (Figure 2A). In some human cancers, this exerts an antiproliferative effect, inhibiting migration and angiogenesis [230], parallel to CR, AAR, and rapamycin [230]. Everolimus alone, or combined with other anticancer agents, has been shown to attenuate cancer cell growth, promote increased cell death, and increase patient survival [221,224,225,231,232]. Sabine et al. [233] observed that patients with early-stage breast cancer had decreased expression of genes involved in cell cycle and proliferation, pyrimidine metabolism, and p53 signaling pathways in response to everolimus. However, the genes involved in the mTOR pathway were not differentially expressed after everolimus treatment, indicating that this compound can modulate other pathways in addition to mTOR [233]. Genes involved in the PI3K/AKT/mTOR pathway are highly responsive to everolimus [234], and as such, everolimus is routinely used in cancer treatments in Europe and the United States. The widespread use of everolimus in clinics highlights the benefits of this compound over rapamycin.

As a derivative of rapamycin, everolimus has also been proposed as a pro-longevity compound and CRM. Everolimus prolongs the survival of HGPS cell models and rescues age-linked cellular defects [227]. In mice, everolimus attenuates neurological decline and vascular dementia [235] as well as decreasing neuronal loss and oxidative stress [236]. Additionally, everolimus reduced amyloid precursor protein (APP) levels, the amyloidogenic Aβ peptide, as well as levels of phosphorylated tau, which are hallmarks of AD pathology [222]. In aged rats, everolimus reversed 37% of age-linked transcriptional changes in kidney tissue, including the reversal of the upregulation of inflammatory response, interferon alpha/gamma response, and apoptosis [226] and, in mice, downregulated glucose metabolism [237] (Table 2). Everolimus also reduces cytokine production [231,238] in liver transplant patients, improves immune function, and prevents immune-induced senescence in elderly humans [223]. In addition to these longevity-promoting effects, everolimus downregulated the protein c-Myc [226]. Increased c-Myc levels are associated with aging [239] and have been reported to respond to rapamycin treatments [240,241] but rarely to CR [242]. c-Myc and SIRT1 function in a negative feedback loop; c-Myc binds the SIRT1 promoter to increase expression, with SIRT1 deacetylating c-Myc to decrease its DNA binding activity [243]. This feedback loop functions independently of mTOR to regulate downstream targets associated with health and lifespan [241]. Furthermore, c-Myc can upregulate ATF4 via GCN2, linking this protein to AAR responses [244]. These complex mechanistic relationships are still under investigation; however, they support the divergence between rapamycin/rapalogs and CR while also highlighting the importance of pathways other than mTOR in regulating the response to longevity-promoting interventions (LPIs).

Both temsirolimus and ridaforolimus have improved solubility and pharmacokinetics over rapamycin [245,246]. Both compounds have been shown to inhibit the growth of cancer cells, with ridaforolimus currently being tested in phase I and II clinical trials. In addition to its potential application as a cancer therapy, temsirolimus ameliorates age-linked phenotypes in a HGPS cell model [247], attenuates tauopathies [248], and clears AD-related Aβ by inducing autophagy in preclinical models [249]. Although promising, information on molecular mechanisms or transcriptomics of these rapalogs is limited.

Rapalogs are associated with numerous beneficial effects linked with healthier aging, such as decreasing inflammation and circulating IGF-1 levels; however, like rapamycin, these derivatives also induce glucose insensitivity. Regardless, these compounds have been documented to reverse age-associated phenotypes in HGPS cell models and ND; benefits that may outweigh potential negative impacts. Notably, most of the research done for these derivatives has been conducted in cancer-based models; therefore, the impact they could have on normal cells/organisms is unclear. Furthermore, the impact of these compounds on genome function is poorly defined, making it difficult to establish whether rapalogs are CRMs; however, although these compounds overlap in their function via inhibition of mTOR, they also likely influence pathways distinct to those altered by CR.

### 5.3. Resveratrol

Resveratrol (3,5,4′-trihydroxystilbene) is a polyphenol produced by plants in response to physiological damage and is found in foods such as grapes, berries, pine nuts, and legumes [250]. Mediterranean diets are rich in resveratrol (as well as many other polyphenols) and have been associated with promoting positive markers of health. For example, the consumption of resveratrol via red wine has been linked to the lower incidence of CVD in France [251] despite high-fat diets. This proposed effect on CVD is due to resveratrol’s antioxidant and anti-inflammatory effects, which have been recapitulated in nonhuman primates [252,253] and have further been associated with the alleviation of age-related diseases [254]. As with other CRMs, resveratrol inhibits cancer cell growth (leukemia [255], breast [256], liver [257], and gastric [258]) and has anticancer effects in multiple animal models ([259,260,261,262,263,264,265]) as well as the capacity to suppress metastasis (reviewed in [266]). Additionally, resveratrol has been documented to decrease age-dependent cognitive decline and AD-like pathologies [267]. In human trials, resveratrol has been shown to regulate neuroinflammation in AD patients [267], improve cognitive function in adults with T2D [268], improve insulin sensitivity [269], and suppress postprandial glucagon [270,271] in overweight individuals. Moreover, in healthy humans, resveratrol improves cerebral blood flow and cognitive performance [272] and decreases IGF-1 levels [273] (Table 3). Since the mentioned pathologies (including cancer and T2D) are often associated with age, resveratrol has been proposed as a CRM and antiaging drug, with evidence demonstrating lifespan increases [274,275,276,277,278,279]. 

Resveratrol generally functions via interaction with, and the activation of, SIRT1, although at higher doses, it also interacts with, and activates, AMPK [280] (Figure 2B). As noted previously, SIRT1 and AMPK feedback to one another in response to CR and proposed CRMs. SIRT1 deacetylates proteins, such as p53, FOXO3a, NF-κB, and peroxisome proliferator-activated receptor gamma coactivator 1α (PGC-1α) through a NAD^+^ substrate that is converted to NAM (reviewed by [281]). Given the role of NAD^+^ as a potent activator of SIRT1, this molecule is key in regulating the energy metabolism and cellular stress mediation effects of resveratrol [279]. To compare the impact of CR to resveratrol, Li and colleagues found that both treatments can upregulate SIRT1, increasing the expression of the pro-longevity protein FOXO3a and the activity of telomerase leading to inhibition of senescence and apoptosis; notably, the impact of resveratrol was greater than that of CR [16]. Additionally, resveratrol reduced the expression of genes encoding inflammatory markers, including IL-6, chemokine (C-C motif) ligand 3 (CCL3), IL-1β, and TNF-α [282] in humans. Analysis of microarray data from breast cancer cells found that 48 h of resveratrol treatment downregulated key genes related to the cell cycle, DNA metabolic process, cellular response to stress, and regulation of cell death [283]. The downregulation of these pathways is similar to that observed in response to CR, maintaining insulin sensitivity [17] and decreasing circulating IGF-1 levels [284]. However, resveratrol was not able to recapitulate the effects of CR in mice [285] and induced differing metabolomes [286]. Limited whole-transcriptome impacts of resveratrol have been generated. In aged mice, resveratrol had minimal effects (note: the mice in question were fasting during treatment, which may impact the interpretation of the data). However, changes in neuron synaptosome function [287] show the potential of resveratrol in treating ND diseases. Resveratrol promotes antioxidant enzyme function, maintains mitochondrial function, and reduces inflammation more than other potential CRMs. Therefore, it is also unlikely an exact mimetic of CR. More information on the impact of resveratrol on the genome, and potential transcription factors regulating these genome-wide changes could provide interesting mechanistic insights into resveratrol’s antiaging properties. 

As with other CRMs, resveratrol can exhibit harmful side effects linked to dosage concentration and duration. For example, in indomethacin-induced gastric ulcerated mice, low-dose resveratrol was protective, whereas higher doses delayed healing [288]. Additionally, resveratrol concentrations that induce cancer cell death are also cytotoxic to healthy cells and tissues (reviewed in [289]). Resveratrol metabolites, produced under certain conditions (e.g., *o*-Quinone), also exhibit detrimental effects, inducing oxidative stress and alkylation [290,291]. It has been suggested that these harmful effects are the result of resveratrol modulating oxidative stress, functioning as an antioxidant or pro-oxidant compound dependent on dosage concentration, duration, and environment. Specifically, high concentrations of resveratrol inhibit DNA repair pathways, triggering ROS production, and inducing cell death (the opposite of which is true for low-dose treatments in promoting health and lifespan) (reviewed in [289]). Further research into the conditions that regulate resveratrol function as a pro- or antioxidant is required to better understand its potential in extending health and lifespan.

### 5.4. Metformin

Metformin (*N*,*N*-dimethylimidodicarbonimidic diamide), derived from the French Lilac (*G. officinalis*), is a commonly used treatment for T2D [296]. Metformin increases AMP/ATP ratios, indicating low cellular energy status and activating AMPK and sirtuin proteins [297]. Surprisingly, despite its widespread use, the exact mechanism by which metformin alters AMP/ATP ratios remains unclear. Metformin likely influences AMPK levels by modulating mitochondrial function and ATP production, triggering AMPK activation and indirectly inhibiting mTORC1 [298], which may parallel CR (Figure 2B). Consequently, the potential of metformin as a CRM relies on its mediation of mitochondrial metabolism and insulin signaling [299,300]. As with CR/AAR and other CRMs, metformin has shown antitumoral effects in various cancers [294,301,302,303,304], neuroprotective effects in ND diseases [305,306], and benefit in treating epilepsy [307]. Metformin also shows potential as an adjuvant cancer therapy [308,309] and is associated with decreased cancer incidence in the general population [310]. Furthermore, metformin has been documented to upregulate longevity-associated phenotypes [311] and extend lifespan in multiple organisms [307,312,313,314,315].

Metformin mediates levels of cytokines linked with inflammation [316,317,318], increases insulin sensitivity [319], and decreases IGF-1 levels [320] (Table 3). This cytokine mediation has been linked to the pro-longevity FOXO3a and the activator protein (AP)-1 transcription factor pathway, both of which can regulate cytokine levels. Interestingly, the ATF2 network was also enriched in response to metformin in human fibroblasts [316], indicating metformin may also function to mimic AAR; therefore, working via multiple pathways to promote longevity. Although cytokine–cytokine receptor signaling was also previously identified in response to rapamycin treatment in normal human fibroblasts, meta-analyses comparing metformin and rapamycin in the same cell line identified divergent transcriptomes [220,316], suggesting that this alteration in cytokine signaling is a downstream impact of these compounds to promote health and lifespan, as opposed to key mechanisms driving response to these proposed CRMs. 

Similar to CR, metformin decreases levels of p53, likely via AMPK-induced activation of SIRT1 function [298]. CR and metformin have common physiological impacts; for example, obese mice under CR and treated with metformin exhibit mitophagy and reduced oxidative stress [298]. Notably, expression of ER stress markers (such as ATF6 and X-box binding protein 1 (XBP-1)) were also decreased. XBP1 has been proposed as a transcription factor for regulating changes in gene expression to CR, whereas ATF6 is linked to the ISR in response to AAR and attenuating global protein translation to counteract cellular stressors. Song et al. [298] also demonstrated that Parkin, a tumor suppressor protein involved in mitophagy, was translocated to the mitochondria in response to CR and metformin in obese mice, inducing autophagy, indicating that both antiaging treatments can lead to the same results albeit by different paths. More research is needed to determine the similarities between metformin and CR mechanisms, at the transcriptomic and epigenomic levels; however, it is unlikely that this compound directly mimics CR. 

## 6. Conclusions

Since discovering the ability of CR to extend the lifespan of rodents, research into CR and CRMs has increased exponentially. In parallel, investigations into the basis of aging have also increased, demonstrating the link between getting older and numerous pathologies (cancer, CVD, T2D, ND). As a result, a plethora of research now demonstrates the ability of CR and CRMs to promote longevity and decrease incidence/delay age-linked diseases. Moreover, it is now clear that CR and various CRMs are able to delay/reverse molecular mechanisms associated with aging processes; however, some CR/CRMs are better at delaying/reversing specific age-linked effects than others. For example, CR and metformin consistently decrease IGF-1 levels and increase insulin sensitivity, whereas resveratrol acts more frequently to promote antioxidant pathways. The complexity of feedback signaling at the cellular and organismal levels indicates that to further pursue mechanisms in promoting longevity, it will be important to consider these effects in future experiments, with current data likely exhibiting controversy due to the lack of the elucidation of feedback loops and time required for these to take effect. 

As whole-transcriptome profiling becomes more widely used, the availability of data regarding the impact of CR, AAR, and CRMs on the genome also increases. These data are beginning to reveal that it is unlikely any of these interventions directly mimic one another, but instead implement divergent mechanisms to arrive at the same endpoint, even when considering the usual factors underlying data variation (species, tissue type, dosage, time). As such, it is important that in the future, researchers are cautious of terminology, and perhaps it is timely to propose that this long-established class of supposed CRMs be referred to as longevity-promoting interventions (LPIs): diets, regimes, and compounds that function to achieve the same goal, health, and lifespan extension, but may do so in divergent manners. Finally, it is well established that CR, AAR, and CRMs (those discussed here, as well as others) extend health and lifespan. Although decreased mTOR function is often used as the benchmark for this, new findings, in part derived from whole-transcriptome analyses, enable the identification of other pathways that may exert influence. Moving forward, examining the crosstalk among these pathways will advance understanding of how to promote healthy aging.

## Figures and Tables

**Table 1:** Summary of the impact of calorie restriction on aging-related pathways of calorie restriction.

	Oxidative stress	Circulating GH/IGF-1	Circulating Glucose/Insulin	Protein Homeostasis/Autophagy	Hormesis/Stress Priming	Protein Translation	Cell Proliferation	Inflammation	Apoptosis	Anti-Cancer	Neuroprotective	Energy Metabolism	Aging	DNA Repair	Immune Response	Other
Caloric Restriction																
Yeast (2% (Non-CR) to 0.5% (CR)) [93]	↓						↓					↓				Transcription Factors (TF): AZF1P, HSF1P and XBP1P
Flies (40 days old. Control: 150 g/L sucrose, 150 g/L autolysed yeast, and 20 g/L agar, *w*/*v* or CR: 33.3% of Control: 50 g/L sucrose, 50 g/L autolysed yeast, and 20 g/L agar, *w*/*v*)) [99]				↑			↓					↓	↓			Upregulated gene TFs: LMX1b; Saal, PCBE, MEF3, PRDM14 Down-regulated gene TFs: DMTF, Zscan1
Mouse (Liver, Heart, Muscle, White Adipose Tissue, Hippocampus, Cortex, Hypothalamus, Cerebellum, Kidney, Lung, Thymus, Spinal Cord, Striatum, Cochlea, Gonad, Colon (Meta-analysis)) [92]	↓							↓		↓						↓ oxidative stress (↓Mt1, Mt2), inflammation (↓Nfκbia, ↓Timp3); ↓tumorigenesis (↓Txnip, ↓Zbt16); ↓metabolism and mRNA splicing (↓Cpsf6, ↓Sfpq, ↓Sfrs, ↓Sfrs18)
Mouse, Rats, Pigs (Meta-analysis) [91]												↑			↓	↑ circadian rhythm, ↓steroid biosynthesis
C57BL/6 Mouse (Male, Muscle, 76% of control, 2 months of age) [87]				↑	↓							↑		↓		
Sprague-Dawley Rats (Male/ Female; Single Cell RNAseq: Brown Adipose Tissue, White Adipose Tissue, Aorta, Kidney, Liver, Skin, Bone Marrow, Aged rats, 70% of Ad libitum from 18 to 27 months) [101]								↓					↓			YBX1- potential molecular switch in CR in adipose derived stem cells of WAT
C57BL/6N Mice (Male, Adipose, 85, 75 and 55% of control for 10 weeks, from 8 weeks of age) [88]		↓										↑			↓	↓transforming growth factor beta and WNT signalling pathways. Mediated by TF: Pax6, Pitx2
C57BL/6, MMTV-TGF-α Mice (Female, Thymus; Chronic Caloric Restriction: 85% of Ad libitum. From 10 weeks to 17/18 weeks) [96]												Δ			Δ	
C57BL/6, MMTV-TGF-α MIce (Female, Thymus; Intermittent Caloric Restriction, 3 weeks Ad libitum, 1 week 60% Ad libitum from 10 weeks to 17/18 weeks) [96]												Δ				
B6C3F1 mice (Male, Hearts, 59% of Ad libitum from 14 months, to 30 months) [89]	↓								Δ	↓		↓	↓		↓	
Flies, (33% yeast/glucose of control) [102]								↓				↓	↓	↓		
Non-human primate (Male, Skeletal Muscle; Adult onset 30% CR, 9 years) [90]					↓				↓			↑	↓			
C3B10RF1 Mice (Female, Liver; 4 weeks CR of 34 month-old mice, 2 weeks 84% Ad libitum 2 weeks 56% Ad libitum) [97]					↓			↓					↓			
C3B10RF1 Mice (Female, Liver; 4 weeks CR, 56% of AL, 7 and 27 months) [97]					↓			↓					↓			
Human (male, female; skeletal muscle, 3–15 years nutrient adequate CR) [94]				↑	↑				↓							
Human (male, PBMC), 3 weeks 30% CR (64–85 years) [98]													↓			↓ olfactory signalling pathways
Human (male, PBMC; 30% CR, 3 weeks (20–28 years) [98]												↓			↓	
Fischer344 Rats, (Male, skeletal muscle 1.3 years, 40% CR) [11]		↓	↓									↑				
Human (Male/female, skeletal muscles; 58.7 ± 7.4, av. 9.6 years of ~30% CR (4–20 years)) [11]		↓					↓	↓								↑ Mitochondrial Biogenesis, mediated by TF FOXO3a/FOXO4
Non-human primate (Male, liver biopsy; 30% decrease in caloric intake compared to control western diet group, between 7–14 years of age) [95]													Δ			Δ changes in xenobiotic pathways

AZF1P: nuclear-localized zinc-finger; HSF1P: heat shock factor; LMX1b: LIM homeobox transcription factor 1-β; MEF3: mitochondrial editing factor 3; PRDM14: PR domain zinc finger protein 14; DMTF: *Drosophila* metal-responsive transcription factor; Zscan1: Zinc finger and SCAN domain-containing 1; Mt: metallothionein; Nfκbia: NFκB inhibitor α; Timp3: metalloproteinase inhibitor 3; Txnip: thioredoxin interacting protein; Zbt16: zinc finger and BTB domain-containing protein 16; Cpsf6: cleavage and polyadenylation specific factor 6; Sfpq: splicing factor proline and glutamine rich; Sfrs: serine- and arginine-rich splicing factor; YBX1: Y-Box 1; Pax6: paired box 6; Pitx2: pituitary homeobox 2; WAT: white adipose tissue.

**Table 2:** Summary of the effects of rapamycin and rapalogs on aging-related pathways.

	Protein Homeostasis/Autophagy	Hormesis/Stress Priming	Protein Translation	Cell Proliferation	Inflammation	Apoptosis	Anti-Cancer	Neuroprotective	Other
Rapamycin									
Mouse Oocyte (100 nm, 2 h) [181]			↓						
Juvenile Human Fibroblasts (2DD; 500 nm, 120 h) [220]	↑		↓	↓					Up-regulation of Cytokine-cytokine receptor signalling, regulated by STAT5A/B TF
Maternally Inherited Leigh Syndrome Human iPS (20 nm, 6 h) [192]	↑		↓	↓					Alleviates mitochondrial defects
Amyotrophic Lateral Sclerosis Mice Spinal Cord (2.33 mg/kg/day, 60 days) [193]	↑		↓	↓					Suppressed immune response/increased mouse survival
Human Prostate Cancer Cell Lines (LNCaP, 22RV1, PC3, DU145; 20 nm, 72 h) [185]				↓	↓	↑			Correlation between Cyclin D1 and rapamycin sensitivity of prostate cancer cells
C57BL/6 mice (endotoxin-uveitis and retinitis induced: retinal inflammation model; 6.0 mg/kg/day) [187]					↓			↑	Decreased NF-kB activity, neuroprotection (decreased rhodopsin)
Wistar Rats (Heart Failure (HF) Model; 1.4 mg/kg/day, from 8 weeks old) [187]	↑		↓			↓			
Sprague-Dawley Rats (Cerebral Ischemia (CI); Kidney and Blood Tissues, Males; 1 mg/kg, 0.5 h prior to CI) [183]	↑				↓	↓			↑ autophagy (↑ BCL-2, ATG13; ↓ULK1); ↓ inflammation (↓TNF-a, IL-1b)
7PA2 cells (APP familial mutation; 0.5 mg/mL, 24 h) [197]	↑		↓					↑	Clearance of ND-linked protein aggregates
3xTg-AD mice (AD model; 2.24 mg/kg, 10 weeks) [197]	↑		↓					↑	Clearance of ND-linked protein aggregates, ↑ autophagy (↑ATG5, ATG7, ATG12)
ND-model (20 nm, 6 h) [192]	↑		↓						Promote energy balance
SAMP8 mice neurons (ND model; 0.5 μM, 72 h) [194]	↑		↓			↓		↑	Clearance of ND-linked protein aggregates,
Humans (0.001% topically. 4 months) [210]									↓ cellular senescence (↓ p16, p21, p53); Decrease in fine wrinkling (↑ collagen VII)
Lmna-/- BAT, WAT (8 mg/kg, every other day) [204]									↓ lipolysis, energy expenditure, fatty acid oxidation; ↑ thermogenesis
**Everolimus**									
Human T1D (1 month before islet transplant; 0.1 mg/kgbw/day) [206]					↓				↓ inflammation (CCL2, CCL3)
Human melanoma cells: Lu1205, WM793 (5 nm, 24 h) [221]				↓					
3xTg-AD mice (AD model) (One dose of 0.167 μg/μL in a volume of 6 μL) [222]								↓	
Elderly Humans (0.5–20 mg/week, 6 weeks) [223]			↓						Enhanced immune response
RT112 and T24 cells (bladder cancer cell models; 0.5–500 nM, 1 h) [224]			↓						↑ AKT phosphorylation (feedback signalling; ↑ GSK3-β phosphorylation)
Post-menopausal women with early breast cancer (5 mg/day, 14 days) [225]				↓			↓		↓ Ki67, S6K1, AKT phosphorylation
HEK293 cells (liver cancer cell model) (1–20 nm, 24 h) [226]			↓						↓ c-Myc
HGPS Fibroblasts (0.1 μM, 2 weeks) [227]	↑								Reversed some cellular aging phenotypes

STAT5A/B: signal transducer and activator of transcription 5; BCL-2: B-cell lymphoma 2; ATG: autophagy-related genes; CCL: C-C motif chemokine ligand; S6K1: ribosomal protein S6 kinase β -1; BAT: brown adipose tissue.

**Table 3:** Summary of the effects of metformin and resveratrol on aging-related pathways.

	Oxidative Stress	Protein Homeostasis/Autophagy	Protein Translation	Cell Proliferation	Inflammation	Apoptosis	Anti-Cancer	Neuroprotective	Other
Resveratrol									
Yeast (2–5 μm) [279]									↑ yeast survival, ↑ SIRT1 activity
Kasumi-1 Xenograft Mice (Leukemia Cell Model; 5–20 mg/kg/day, 24 days) [255]						↑	↑		
Kasumi-1 (Leukemia Cell Model; 50 μm, 6 h) [255]						↑	↑		
MCF-10A-Tr xenograft mice (breast cancer animal model (40 mg/kg/day, 30 days) [261]	↓				↓	↑	↑		Decreased base excision repair
AD Patients (500–2000 mg/day, 52 weeks) [267]					↓				↓ inflammation (IL-8, IL-1R4, IL-12P40, IL-12P70); ↓ permeability to inflammatory agents (↓ MMP9); maintained cerebral spinal fluid proteins
AβPP/PS1 mice (AD animal model; 16 mg/kg/day, 10 months) [292]									Improved synaptic activity (increased synaptophysin) Decreased protein aggregates (BACE, ADAM 10) Regulated by AMPK signalling (Increased AMPK and LKB1 phosphorylation, decreased p53 acetylation)
TCDD CYP1A induced expression induced in MCF-10A cells (breast cancer cell model; 5–50 μM, 3 days) [293]	↓								Decreased oxidative DNA damage (CYP1A1, CYP1B1)
**Metformin**									
HepG2 cells (liver cancer model; 1–10 mM, 24 h) [294]				↓		↑			↑caspase 3, ↑AKT phosphorylation
HepG2 xenograft mice fed with 60% high-fat diet (250 mg/kgbw/day, 4 weeks) [294]				↓					↓Cyclin D1, ↑hypoxia induction and maintenance of micro vessel density: ↑ CA-9, ↓VEGFR
Bone marrow mice -derived macrophages (BMDMs; 2 mM, 24 h) [295]					↓				
High fat-fed C57B6L male mice (300 mg/kgbw/day-11 weeks) [295]					↓				↓IL-6, ↓TNF-α

MMP9: matrix metallopeptidase 9; BACE: beta-secretase 1; ADAM10: a disintegrin and metalloproteinase domain-containing protein 10; CYP1: cytochrome P450; CA-9: carbonic anhydrase 9; VEGFR: Vascular endothelial growth factor receptor; CK19: cytokeratin-19, α-SMA: alpha smooth muscle actin; GLUT1: glucose transporter 1; GREB1: growth-regulating estrogen receptor binding 1; AP-1: activator protein 1; ARE: antioxidant response elements; SOD: superoxide dismutase; CAT: catalase; GHS: glycinyl-histidinyl-serine; BDNF: brain-derived neurotrophic factor; ER: endoplasmic reticulum.

## Abbreviations

AAR	Amino acid restriction
AARE	Amino acid response element
AD	Alzheimer’s disease
ADAM10	Disintegrin and metalloproteinase domain-containing protein 10
AP-1	Activator protein 1
APP	Amyloid precursor protein
AKT	Protein Kinase B
AMP	Adenosine monophosphate
AMPK	Adenosine monophosphate kinase
APP	Amyloid precursor protein
ARE	Antioxidant response elements
Arf-1 GTPase	ADP ribosylation factor
α-SMA	Alpha smooth muscle actin
ATF	Activating transcription factor
ATG	Autophagy-related genes
ATP	Adenosine triphosphate
AZF1P	Nuclear-localized zinc-finger
BACE	Beta-secretase 1
BAT	Brown adipose tissue
BCAA	Branched-chain amino acids
BCL-2	B-cell lymphoma 2
BCL-XL	B-cell lymphoma-extra-large
BDNF	Brain-derived neurotrophic factor
CA-9	Carbonic anhydrase 9
CamKKB	Calcium/calmodulin-dependent protein kinase kinase B
CASTOR	Cytosolic arginine sensor for mTORC1
CAT	Catalase
CCL3	Chemokine (C-C motif) ligand 3
Cpsf6	Cleavage and polyadenylation specific factor 6
CK19	Cytokeratin-19
CR	Caloric restriction
CRMs	Caloric restriction mimetic
CVD	Cardiovascular disease
CYP1	Cytochrome P450
DMTF	*Drosophila* metal-responsive transcription factor
eIF2	Eukaryotic initiation factor 2
ER	Endoplasmic reticulum
FGF21	Fibroblast growth factor 21
FOXO3a	Forkhead box O3a
GADD34	Growth arrest and DNA damage-inducible protein 34
GATOR	GTPase-activating protein toward rags
GCN2	General control nonderepressible 2
GDP	Guanosine diphosphate
GHS	Glycinyl-histidinyl-serine
GLUT1	Glucose transporter 1
GREB1	Growth-regulating estrogen receptor binding 1
GSK-3β	Glycogen synthase kinase-3β
GTP	Guanosine triphosphate
HGPS	Hutchinson–Gilford progeria syndrome
HIF1α	Hypoxia-inducible factor-1α
HSF1P	Heat shock factor
IGF-1	Insulin-like growth factor-1
IL	Interleukin
IRS-1	Insulin receptor substrate 1
ISR	Integrated stress response
JNK	C-jun N-terminal kinase
LKB1	Liver kinase B1
LMX1b	LIM homeobox transcription factor 1-β
LPIs	Longevity-promoting interventions
MAPK	Mitogen activator protein kinase
MEF3	Mitochondrial editing factor 3
MMP9	Matrix metallopeptidase 9
Mt	Metallothionein
mTOR	Mammalian target of rapamycin
mTORC1	Mammalian target of rapamycin complex 1
mTORC2	Mammalian target of rapamycin complex 2
NAD^+^	Nicotinamide adenine dinucleotide
NAM	Nicotinamide
ND	Neurodegenerative diseases
NF-κB	Nuclear factor-κB
Nfκbia	NFκB inhibitor α
NRF2	Nuclear receptor factor 2
Pax6	Paired box 6
PDK1	3-phosphoinositide-dependent protein kinase 1
PGC-1α	Peroxisome proliferator-activated receptor gamma coactivator-1α
PI3K	Phosphoinositide 3-kinase
Pitx2	pituitary homeobox 2
PRDM14	PR domain zinc finger protein 14
RagA/B	Ras-related GTP-binding protein A and B
RagC/D	ras-related GTP-binding protein C and D
Rheb	RAS homolog enriched in brain
ROS	Reactive oxygen species
S6K1	Ribosomal protein S6 kinase
SAAR	Sulfur amino acid restriction
SAM	S-adenosyl-methionine
SAMTOR	SAM sensor upstream of mTORC1
Sfpq	Splicing factor proline and glutamine rich
Sfrs	Serine- and arginine-rich splicing factor
SIRT1	Sirtuin 1
SOD	Superoxide dismutase
SPD	Starvation pseudodiabetes
STAT3	Signal transducer and activator of transcription 3
STAT5A/B	Signal transducer and activator of transcription 5A/B
T2D	Type 2 diabetes
Timp3	Metalloproteinase inhibitor 3
TNFα	Tumor necrosis factor-α
TSC	Tuberous sclerosis complex
Txnip	Thioredoxin interacting protein
ULK1	Unc-51 Like Autophagy Activating Kinase 1
VEGFR	Vascular endothelial growth factor receptor
WAT	White adipose tissue
WNT	Wingless-related integration site
XBP-1	X-box-binding protein 1
YBX1	Y-Box 1
Zbt16	Zinc finger and BTB domain-containing protein 16
Zscan1	Zinc finger and SCAN domain-containing 1
